# Ouroboros: cross-linking protein expression perturbations and cancer histology imaging with generative-predictive modeling

**DOI:** 10.1093/bioinformatics/btae399

**Published:** 2024-09-04

**Authors:** Srijay Deshpande, Sokratia Georgaka, Michael Haley, Robert Sellers, James Minshull, Jayakrupakar Nallala, Martin Fergie, Nicholas Stone, Nasir Rajpoot, Syed Murtuza Baker, Mudassar Iqbal, Kevin Couper, Federico Roncaroli, Fayyaz Minhas

**Affiliations:** Department of Computer Science, Tissue Image Analytics Centre, University of Warwick, Coventry, CV4 7AL, United Kingdom; Manchester Academic Health Science Centre, Faculty of Biology, Medicine and Health, University of Manchester, M13 9PL, Manchester, United Kingdom; Manchester Academic Health Science Centre, Faculty of Biology, Medicine and Health, University of Manchester, M13 9PL, Manchester, United Kingdom; Manchester Academic Health Science Centre, Faculty of Biology, Medicine and Health, University of Manchester, M13 9PL, Manchester, United Kingdom; Division of Neuroscience and Experimental Psychology, University of Manchester, Manchester, M13 9PL, United Kingdom; School of Physics and Astronomy, University of Exeter, Exeter, EX4 4QL, United Kingdom; Division of Informatics, Imaging and Data Science, University of Manchester, Manchester, M13 9PT, United Kingdom; School of Physics and Astronomy, University of Exeter, Exeter, EX4 4QL, United Kingdom; Department of Computer Science, Tissue Image Analytics Centre, University of Warwick, Coventry, CV4 7AL, United Kingdom; Manchester Academic Health Science Centre, Faculty of Biology, Medicine and Health, University of Manchester, M13 9PL, Manchester, United Kingdom; Manchester Academic Health Science Centre, Faculty of Biology, Medicine and Health, University of Manchester, M13 9PL, Manchester, United Kingdom; Manchester Academic Health Science Centre, Faculty of Biology, Medicine and Health, University of Manchester, M13 9PL, Manchester, United Kingdom; Division of Neuroscience and Experimental Psychology, University of Manchester, Manchester, M13 9PL, United Kingdom; Department of Computer Science, Tissue Image Analytics Centre, University of Warwick, Coventry, CV4 7AL, United Kingdom

## Abstract

**Summary:**

Imagine if we could simultaneously predict spatial protein expression in tissues from their routine Hematoxylin and Eosin (H&E) stained images, and create tissue images given protein expression profiles thus enabling virtual simulations of how protein expression alterations impact histology in complex diseases like cancer. Such an approach could lead to more informed diagnostic and therapeutic decisions for precision medicine at lower costs and shorter turnaround times, more detailed insights into underlying disease pathology as well as improvement in predictive and generative performance. In this study, we investigate the intricate correlation between protein expressions obtained from Hyperion mass cytometry and histopathological microstructures in conventional H&E stained glioblastoma (GBM) samples, unveiling morphological patterns and cellular-level spatial alterations associated with protein expression changes. To model these complex relationships, we propose a novel generative-predictive framework called Ouroboros for producing H&E images from protein expressions and simultaneously predicting protein expressions from H&E images. Our comprehensive sample-independent validation over 9920 tissue spots from 4 GBM samples encompassing visual image analysis, quantitative analysis, subspace alignment and perturbation experiments shows that the proposed generative-predictive approach offers significant improvements in predicting protein expression from images in comparison to baseline methods as well as accurate generation of virtual GBM sample images. This proof of concept study can contribute to advancing our understanding of histological responses to protein expression perturbations and lays the foundations for further developments in this area.

**Availability and implementation:**

Implementation and associated data for the proposed approach are available at the URL: https://github.com/Srijay/Ouroboros.

## 1 Introduction

The evolution of spatial gene and protein expression techniques has revolutionized biomedical research, opening avenues for systematic exploration of histomic, transcriptomic and proteomic profiles and their “cross-omics” interactions (He *et al.* 2020, Dawood *et al.* 2021). Such approaches offer more holistic understanding of tissue microenvironment for various diseases such as cancer, their growth trajectories and potential drug sensitivities. In contrast to conventional “bulk” sequencing, spatial gene and protein expression profiling provides more localized and detailed insights into the underlying pathology (Li and Wang 2021). Spatial transcriptomics offer high-throughput measurement of gene expression at a *spot*-level resolution in comparison to complementary approaches such as FISH (Moses and Pachter 2022). Spatial protein expression measurement techniques such as Imaging Mass Cytometry (Giesen *et al.* 2014), available through the Hyperion imaging system (https://www.standardbio.com/products/instruments/hyperion), allow for characterization of the underlying protein expression of multiple markers at the cellular level. This overcomes the limitations of traditional methods that focus primarily on gene expression and provides a more direct and precise analysis of protein dynamics in various biological contexts (Rochais *et al.* 2022).

The integration of routine H&E stained whole slide images (WSIs)—the bedrock of clinical and diagnostic pathology across the globe—with spatial gene and protein data facilitates exploring the link between image patterns and tissue’s molecular state. Utilizing these multi-modal datasets alongside recent machine learning advances enables a deeper understanding of tissue morphology and molecular characteristics across diseases, including cancer, while addressing issues associated with high cost of spatial molecular analyses.

Previous work in this domain has focused on using bulk sequencing data to cross-link WSIs with transcriptomics and mutational profiles through machine learning methods that can predict key mutations, molecular pathways, gene expression and transcriptomic states (Kather *et al.* 2020, Bilal *et al.* 2021) with the main goal being triaging of cases for sequencing for effective case management and therapeutic decision-making with shorter turnaround times at lower costs. The utilization of bulk sequencing data presents a limitation in accurately correlating imaging patterns with underlying expression profiles with such approaches. This challenge has been partially addressed by recent studies, which have used machine learning techniques to predict localized gene expression profiles based on WSI data (He *et al.* 2020, Dawood *et al.* 2021). While these techniques offer valuable insights into the relationship between spatial or bulk gene expression and imaging characteristics, they are not equipped to directly determine the impact of altering specific gene or protein expressions on tissue morphology.

To the best of our knowledge, current methods that aim to cross-link omics data with conventional WSI predominantly focus on predictive modeling (Park *et al.* 2022). These methods can predict a range of factors related to direct or indirect spatial expression characteristics. However, they fall short of generating synthetic tissue images that reflect alterations in the underlying spatial expression profiles. Generation of synthetic tissue images from protein expression can enable exploration of effects of protein expression changes thus leading to a more in-depth understanding of cancer biology. For example, in aggressive glioblastomas (GBMs), such an approach could help elucidate the impact of microglia-specific genes on cellular morphology and histology. Other potential utilities include improved cancer diagnosis, biomarker discovery, personalized medicine, monitoring disease progression and treatment response as well as providing insights for discovery of novel drugs (Ohno *et al.* 2014). For example, accurate predictions of changes in expression or mutations in a biopsy sample of brain tissue could help accurately diagnose GBM and, consequently, alter a patient’s treatment pathway. We also hypothesize that a machine learning approach that can predict protein expression from images while simultaneously being capable of generating such images can give higher accuracy in protein expression prediction. This is a crucial gap highlighting the need for advanced machine learning techniques capable of simulating changes in tissue morphology based on modifications in spatial protein expression profiles. Although there has been work on studying the effect of chemical perturbations on single-cell level changes (Kana *et al.* 2023, Palma *et al*. 2023), the impact of protein expression perturbations at the tissue level has not been explored especially in the context of routine H&E imaging. We are not aware of any existing method that can jointly predict protein expression and generate images concurrently, especially in the context of establishing a connection between H&E tissue images and protein expression profiles.

**Figure 1.**
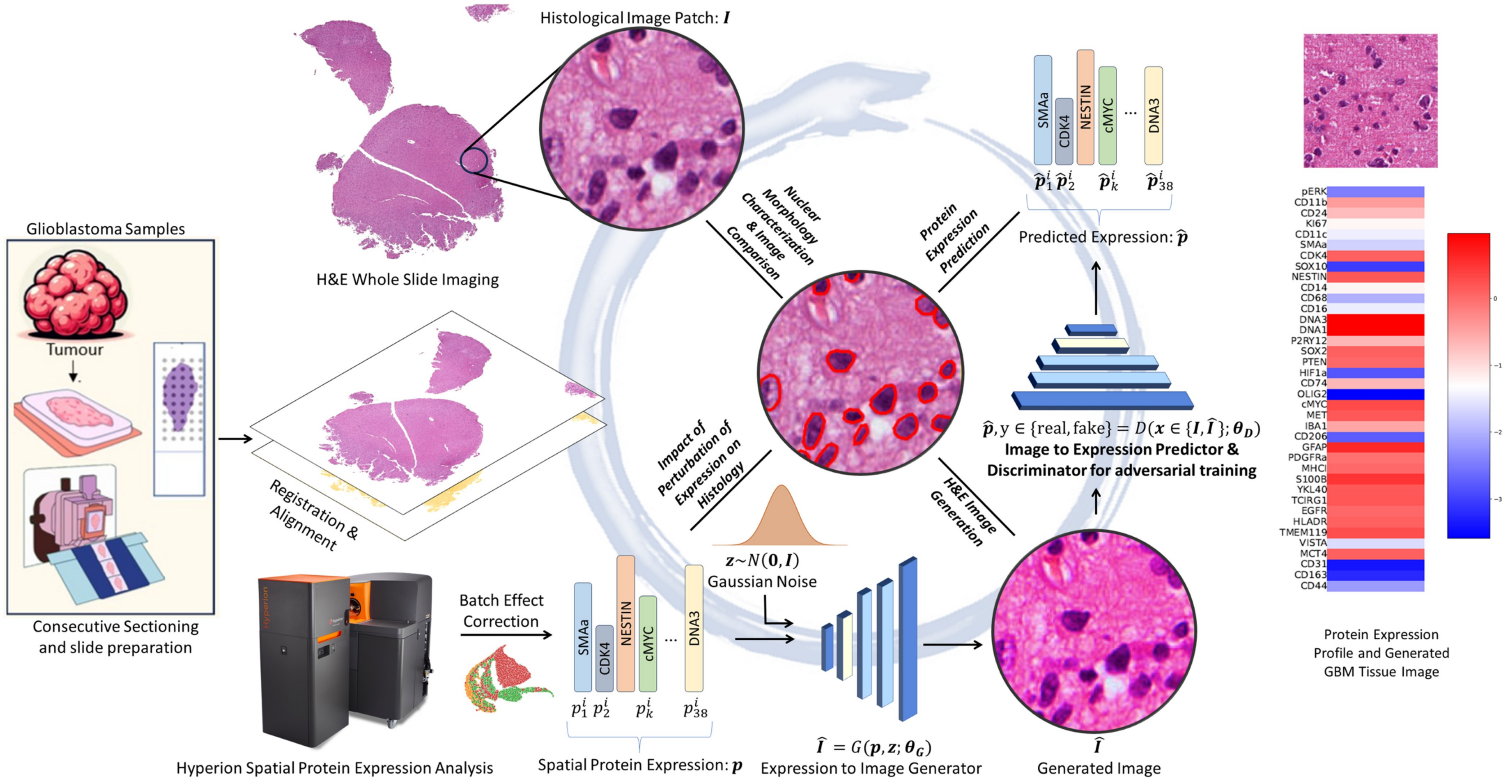
The Ouroboros pipeline allows simultaneous generation of virtual H&E images from protein expression and prediction of spatially localized protein expression from H&E images. For this purpose, two consecutive sections of glioblastoma (GBM) samples are obtained. One section is H&E stained for whole slide imaging and the other is used for measurement of spatial expression profiles of 38 different protein markers through the Hyperion system. After preprocessing (alignment/registration, patch-level expression averaging, and batch effect correction of protein expression data), adversarial training is used to simultaneously train an image generator and a discriminator/predictor. The generator can generate synthetic H&E images from the given protein expression profile whereas the discriminator cannot only discriminate between real and fake/synthetic images given to it as input but can also predict the expression of proteins in the given input patch. On top of generating H&E images from protein expression and predicting protein expression from H&E images, this pipeline allows us to analyze the association between protein expression and morphological features of histology and to study the effects of perturbing protein expression on tissue morphology.

In this proof-of-principle study, we propose a novel generative-predictive pipeline called *Ouroboros* for simultaneously predicting spatial protein expression from routine histological imaging as well as generating such images from spatial protein expression profiles (see Fig. 1) (The proposed generative-predictive framework is inspired by the ancient symbol of a serpent consuming its own tail.). We have chosen to use Glioblastoma (GBM) samples due to their high heterogeneity and histological complexity (Inda *et al.* 2014, De Bacco *et al.* 2023).

The major contributions of this work are listed below:

1. To the best of our knowledge, this is the first proof of principle study that explores correlations between spatial protein expressions and histological patterns through a generative-predictive pipeline.
2. We demonstrate that the proposed approach offers improved predictive performance when predicting protein expression from routine H&E WSIs in comparison to baseline methods for glioblastoma (GBM) samples.
3. The images generated by our framework are realistic in terms of their Frechet Inception Distance as well as nuclear morphological characteristics.
4. We demonstrate that the proposed approach can effectively capture the effects of perturbations in protein expression on whole slide images.

## 2 Materials and methods

Figure 1 provides an overview of the Ouroboros framework. The methodology uses consecutive sections of glioblastoma (GBM) samples stained with H&E, coupled with the expression profiles of 38 distinct protein markers acquired through the Hyperion system. After initial pre-processing steps involving alignment/registration, patch-level expression averaging, and batch effect correction of protein expression data, adversarial training is used to concurrently train an image generator and a discriminator which also acts as a predictor of protein expression. Consequently, the framework can generate tissue images based on protein expressions and predict protein expressions from the histology images.

The protein expression profile serves as an input to the image generator network for the creation of histology images. The Generative Adversarial Network (GAN) based network incorporates a discriminator, which not only assesses the realism of generated images but also predicts the protein expression in them. The generator and the discriminator engage in a competitive process: The generator creates synthetic images while the discriminator evaluates whether the generated data is real or fake. The generator aims to produce data that is indistinguishable from real examples, while the discriminator strives to correctly classify between real and generated data. As training progresses, both networks improve their performance, and the adversarial interplay between the generator and discriminator drives the network to generate high-quality, realistic outputs.

Beyond the generation of H&E images from protein expression and the prediction of protein expression from images, this pipeline enables the analysis of the association between protein expression and nuclear morphological features in histology. In addition, it facilitates the exploration of the effects of perturbing protein expression on the tissue.

Below, we discuss various steps in the pipeline in detail.

### 2.1 Dataset

We acquired four GBM samples from four different patients at the University of Manchester. Consecutive sectioning was performed to obtain spatial protein expression measurements of 38 different protein markers using the Hyperion system on one section with a matching H&E stained whole-slide image (WSI) from the consecutive section. The size of each WSI is approximately 16 000×16 000 pixels with a resolution 0.43 µm/pixel. For training and validating the Ouroboros framework, we extract 9920 image patches of size 256 × 256 from whole-slide images with each patch centered on the location of a 128 × 128 pixel spot for which protein expression data is available. The number of patches from the four images are 2894, 1145, 4129, and 1753. Choosing larger patches in comparison to spots enables more context aware image generation as well as offsets the effects of any misalignment in registration.

Tissue sections (5 µm thickness) underwent staining with lanthanide-conjugated antibodies as instructed by manufacturer (https://www.standardbio.com/products/instruments/hyperion), and were imaged on a Hyperion imaging mass cytometer. The resolution of the resulting images is 1 µm/pixel. Staining was reviewed by a neuropathologist using MCD Viewer (Standard BioTools). Shot noise and hot pixels were removed from the resulting data using the IMC-Denoise algorithm (Lu *et al.* 2023).

### 2.2 Data preprocessing

After obtaining protein expression and WSI data of consecutive sections, we performed registration to align the protein expression data and the H&E image using HALO (https://learn.indicalab.com/courses/image-analysis-platform/lessons/halo-video-tutorials/topic/08-image-registration/). It is important to note that this process does not lead to exact cell-level alignment between consecutive. Consequently, each whole-slide images is then partitioned into a grid of spots, each measuring 128 × 128 pixels. The protein expression profile consisting of expression counts of 38 protein markers for a given spot is obtained by averaging cell-level protein expressions in the corresponding hyperion image.

**Figure 2.**
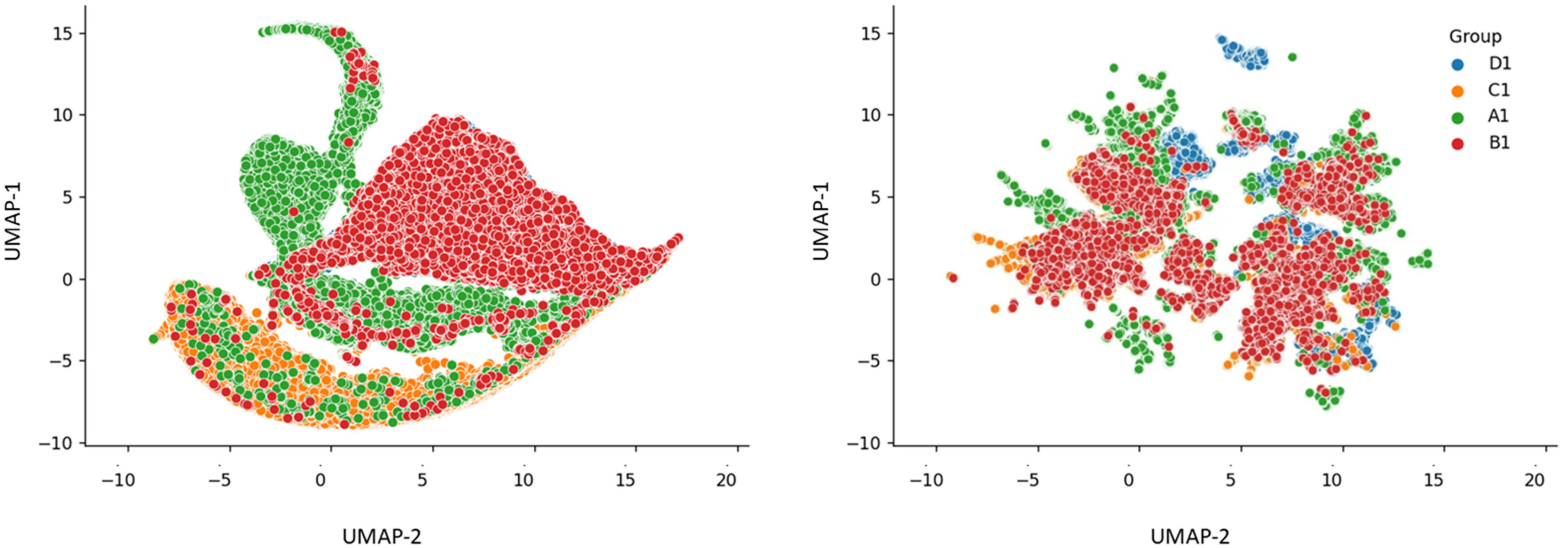
UMAP plots depicting protein expression profiles across GBM whole slide images. The left plot illustrates UMAP 2D points calculated from the original protein expression profiles, while the right plot showcases UMAP points derived from protein expressions after data harmonization.

After acquiring spot-level protein counts, we log-transformed nonzero values and applied z-scoring. Zero counts were assigned a markedly lower value (by a factor of 1000 in terms of original expression), distinguishing them as a separate category from positive expressions. This allows for interpretation of expression levels as either above or below the average across the whole dataset. In order to harmonize protein expression data for the four patients, we used pyComBat for bias effect correction (Zindler *et al.* 2020). Subsequently, we generated a UMAP plot of the protein expression data to analyze the effects of this adjustment (see Fig. 2). This representation illustrates the anticipated phenomenon of clustered protein expression values overlapping after data harmonization.

### 2.3 Spot-level protein expression profiles

The protein expression profile for a given patch or spot is a 38D vector that includes the expression values of 38 distinct protein biomarkers, such as SMAa, CDK4, NESTIN, etc. as well as metal-based nuclear markers such as DNA1 and DNA3. We represent this protein expression vector as **p**. This allows us to represent each whole slide image $W_{j},j=1,2,3,4$ in our dataset as $W_{j}=\{(p_{i}^{j},I_{i}^{j}),i=1\ldots N_{j}\}$ each comprising of *N_j_* patches with each patch or spot consisting of a protein expression vector $p_{i}^{j}$ and a corresponding image $I_{i}^{j}$.

### 2.4 Histological image generation

We aim to model the conditional generation of histology images based on the protein expression profiles and also enable protein expression prediction from histology images. To achieve this, our approach utilizes a generative adversarial network (GAN) architecture capable of producing high-quality histology images based on protein expression profiles. In this GAN, the generator *G* is used for generating histology images from protein expression profiles, while the discriminator *D* serves the dual purpose of assessing the realism of tissue images and providing predictions for the associated protein expression profiles.

Taking inspiration from BigGAN (Brock *et al.* 2019) for its design, the generator accepts input noise *z* and a protein expression vector **p** for generating a histology image $\hat{I}$ of size 256 × 256 pixels. The process is represented as $\hat{I}=G(p,z;\theta_{G})$, where *θ_G_* represents the trainable weight parameters within the generator. Introducing Gaussian noise as input adds variability to the generated images. Supplementary Fig. S1 shows the structure of generator and its use of residual blocks and nonlocal self-attention blocks [see Brock *et al.* (2019) for details] to capture finer details in tissue images as well as up-sampling layers to double input dimensionality. This approach ensures that the histology image is generated by considering both the protein expression vector and additional noise for introducing variability.

### 2.5 Protein expression prediction

Our proposed discriminator *D* extends the original BigGAN (Brock *et al.* 2019) discriminator to not only output the realism score of the image but also predict the spatial protein expression: $\hat{p}=D(\hat{I};\theta_{D})$, where *θ_D_* represents the trainable parameters of the discriminator. The architecture of the discriminator used in Ouroboros framework is shown in Supplementary Fig. S1. This dual functionality of the discriminator accomplishes two primary objectives within the overall pipeline: (i) generating a tissue image from protein expression and (ii) predicting protein expression from the image.

### 2.6 Loss function terms

The different loss components used while training the network are described below:

**Image reconstruction loss**: This term quantifies the reconstruction error between the ground truth **I** and the generated tissue image $\hat{I}$ using the mean absolute error (MAE) between the two and is written as: $L_{I}(I,\hat{I};\theta_{G})=\text{MAE}(I,\hat{I})$.

**Protein expression reconstruction loss**: This term measures the reconstruction error between the ground truth protein expressions **p** (used as input) and the protein expression profile predicted from a generated image $\hat{p}$ using mean square error (MSE) loss: $L_{r}(p,\hat{p};\theta_{G})=\text{MSE}(p,\hat{p})$

**Protein expression prediction loss**: This term measures the reconstruction error between the ground truth protein expressions **p** (used as input) and the protein expression profile predicted from a real image $\hat{p}$ using mean square error (MSE) loss: $L_{p}(p,\hat{p};\theta_{D})=\text{MSE}(p,\hat{p})$.

**Adversarial loss terms**: We use an adversarial loss function (Goodfellow *et al.* 2014) for both discriminator and generator in the Ouroboros framework. The adversarial min-max loss function is given by,

(1) $$\begin{array}{c} \underset{\theta_{G}}{\min}\underset{\theta_{D}}{\max}L_{\mathrm{GAN}}(I,p,z;\theta_{G},\theta_{D})=E_{I∼P_{I}}[\text{log}\;D(I;\theta_{D})]\\ +\;E_{p∼P_{p},z∼N(0,I)}[\text{log}(1-D(G(p,z;\theta_{G});\theta_{D})] \end{array}$$

where *P_I_* and *P_p_* represent the probability distributions over tissue images and protein expressions, respectively. Thus, the overall learning problem is formulated as an adversarial optimization problem based on the linear combination of the above loss terms. The framework is trained by minimizing the objective $L=L_{I}+\lambda_{1}L_{r}+\lambda_{2}L_{p}+\lambda_{3}L_{\mathrm{GAN}}$, where $\lambda_{1},\lambda_{2}$, and *λ*_3_ represent the weights of corresponding loss components.

### 2.7 Model training and validation

Over a given training set of images and their spot level expression profiles, the framework uses Adam optimization with an initial learning rate of $10^{-4}$, initial momentum of 0.5, and a batch size of 16. During training, we set the coefficients for weighing the loss components as follows: $\lambda_{1}=1,\;\lambda_{2}=1,\;\lambda_{3}=0.01$.

Performance assessment is done through a leave-one-patient-out cross-validation protocol in which we keep patches from three WSIs for training and use patches from the held-out WSI for testing, and repeat the process four times by holding out each WSI in turn. In the subsequent section, patches used for training are referred to as “train patches” and those utilized for testing are denoted as “test patches.” Performance metrics are evaluated over test patches. It must be noted that for quantitative analysis, we crop out the central 128 × 128 pixel portion from generated images to align with spot sizes for which protein expression data is available.

We assess the quality of generated images using the widely used Frechet Inception Distance (FID) (Heusel *et al.* 2017). This metric quantifies the model’s ability to reproduce the original data distribution with a lower FID score indicating higher quality in image generation. To evaluate the protein expression prediction performance of the Ouroboros framework, we utilize Pearson’s correlation and Spearman’s correlation between true and predicted expression values as metrics.

To assess the concordance between nuclear morphological features of cells in true and synthetic images, we computed the morphological feature distance between morphological features extracted from both real and synthetic images. For this purpose, nuclei are first detected in an H&E image using the StarDist algorithm (Schmidt *et al.* 2018) in QuPath software (Bankhead *et al.* 2017) followed by extraction of 63 different morphological features for each detected cell which are then averaged for all cells in the patch. These morphological features capture information about size, solidity, H&E distribution, etc.

## 3 Experiments and results

### 3.1 Visual results

**Figure 3.**
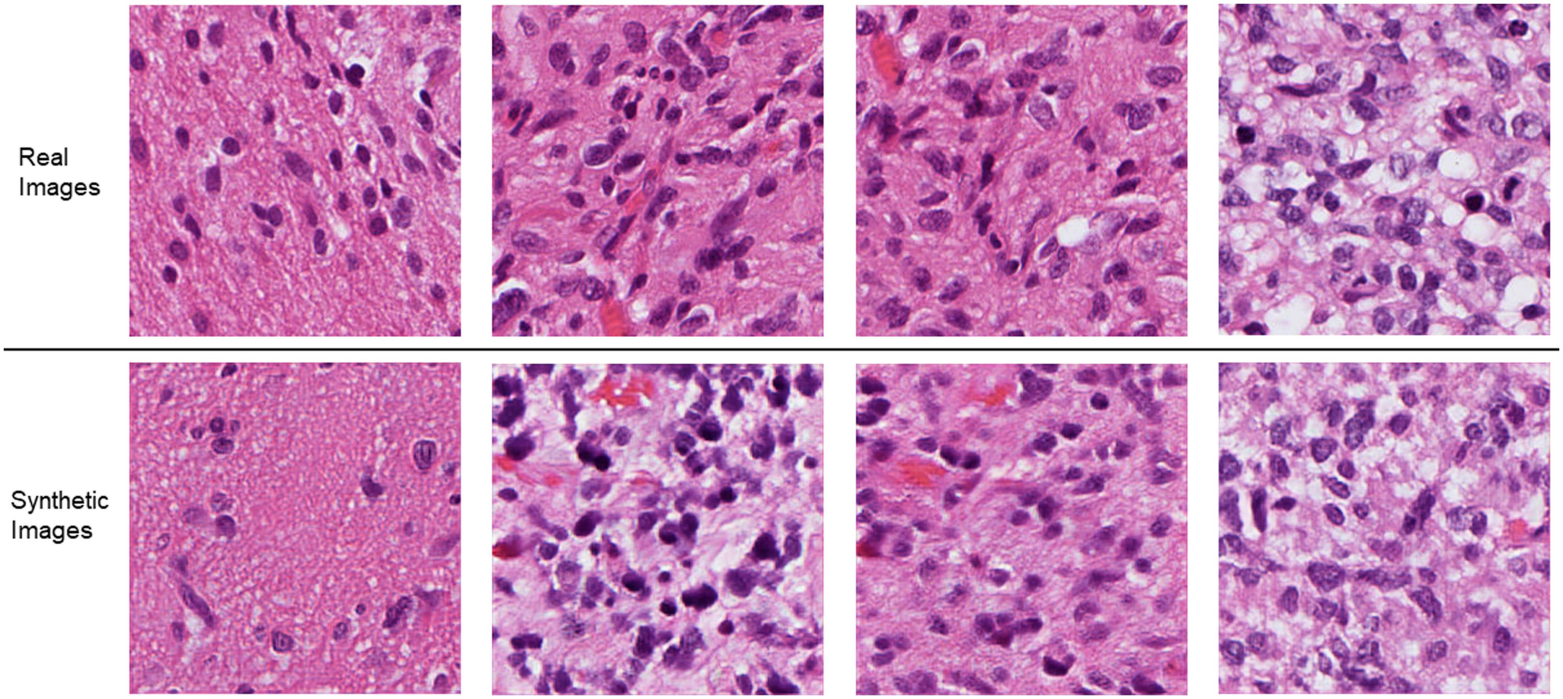
Representative examples of real (top row) and generated images (bottom row) from protein expression profiles in testing.

Figure 3 showcases real image patches from whole-slide GBM images along with their synthetic counterparts generated from protein expression data in testing. High fidelity in cellular features and overall texture is apparent in the generated images. It is important to note that as the generated images are based solely on protein expression values and no cellular layout information is used, the arrangement of cells in the generated patches can differ from true patches taken from the same area. However, it is important to note the consistency of overall tissue structure and cellular/nuclear morphologies.

### 3.2 Quantitative evaluation of image generation

In order to quantify the degree of concordance between true and generated images, we compute the Frechet Inception Distance (FID) and morphological feature distance between regions of 128 × 128 pixels extracted from real test patches and their synthetic counterparts, as these regions correspond to protein expression profiles. For the purpose of establishing a baseline for the FID for this data, we compare the FID between synthetic and real patches with the FID between synthetic patches and random noise of the same 128 × 128 pixel dimensions. The FID score of 69.17 ± 0.89 for synthetically generated images in comparison to random noise images exhibiting FID of 489.42 ± 5.76 indicates high-quality generation.

For assessment of the alignment between morphological features of cellular nuclei in true and synthetic images, we calculated the morphological feature distance between each real and its corresponding synthetic image and compared it to the average morphological feature distance of the real image to 100 randomly sampled real images from the same WSI followed by one-sided Wilcoxon signed-rank test over the paired distances to establish statistical significance. For test patches, the average morphological feature distance for images generated through the Ouroboros framework is 5.6, which is significantly <6.9 obtained for the paired baseline (Wilcoxon *P*-value <10^−10^). These observations highlight the fact that the generated image patches preserve nuclear morphological features.

### 3.3 Protein expression prediction results

**Figure 4.**
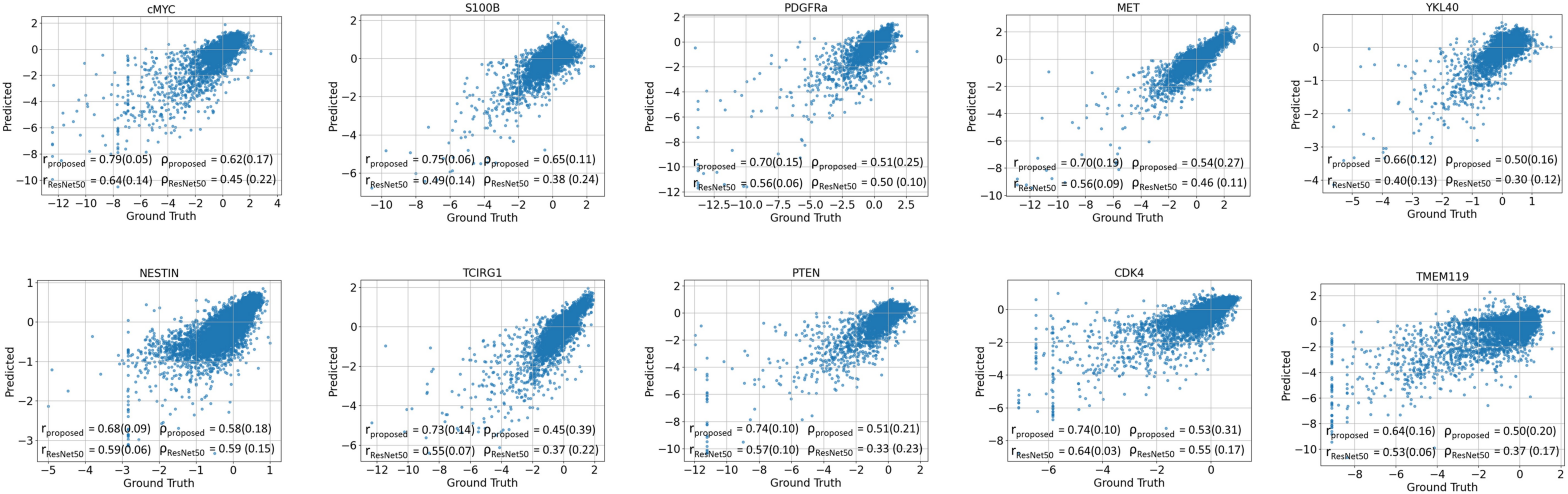
Scatterplots of ground truth versus predicted protein expression profiles for top predicted protein biomarkers. Also shown is the comparative performance of Ouroboros versus the baseline ResNet50 model in terms of the mean (standard deviation) of Pearson (*r*) and Spearman correlation coefficients (*ρ*) between predicted and actual expression of various proteins across test image patches (correlation *P*-value ≪0.001).

Figure 4 shows the correlation between true and predicted expression scores for different proteins over test patches. For a comparative baseline, we trained a ResNet50 model (He *et al.* 2016) to predict protein expression from image patches and validated it using the same leave-one-patient-out cross-validation protocol discussed in Section. The Ouroboros framework demonstrates marked enhancement over baseline results, substantiating our initial hypothesis that generative-predictive pipelines can outperform prediction-only methods in protein expression prediction (see Supplementary Table S1 for detailed results over all proteins).

### 3.4 Expression perturbation and interpolation experiments

**Figure 5.**
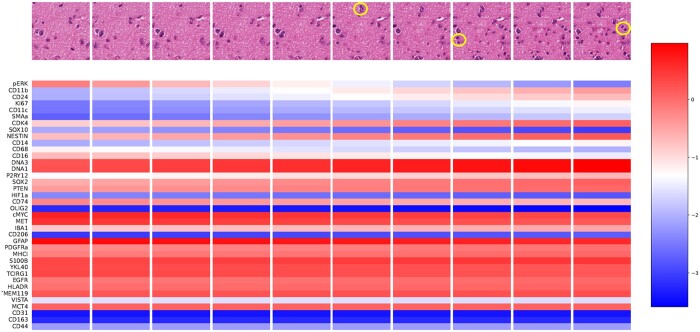
Results of interpolation experiment. Protein expression values and corresponding synthetic images generated in testing across 10 linear interpolation steps (left to right). New emerging cells after protein expressions perturbations are circled in yellow color.

In order to assess if the proposed methodology can be used for assessing the impact of protein expression perturbations on tissue images, we conducted a simple experiment in which we linearly interpolate between the distinct expression values of two selected spots in a WSI, generating ten intermediate protein expression vectors. For each vector, we generated a corresponding patch using our generative method trained on other WSIs. The resulting patches and their associated protein expression values for the 10 interpolation steps are shown in Fig. 5. Based on pathologist review, it can be noted that the number of cells increases from left to right with the last four synthetic patches being highly cellular as opposed to other patches. Images associated with steps 2–6 show a neoplastic cell on the left likely due to high expression of oncoproteins cMYC and MET. Increased CD68, CD74, IBA1, and PYR12 levels are expected to be associated with the presence of small cells (possibly macrophages) in the first image. The values of SOX2 across all steps suggest some stemness, either glioma stem cells or more likely tumor cells with stem cell phenotype considering low expression of SOX10 and NESTIN. All generated patches show the same fibrillary background, which can be attributed to GFAP expression. In summary, the generated images reflect closely the changes expected from such protein expression changes with lymphocytes, microglial cells, macrophages and tumor cells disappearing and appearing in the images depending on the values of proteins defining their phenotype. It is important to note that this result was not selectively chosen but rather representative, showcasing the promise and potential of our approach. This underscores the method’s robustness and its ability to yield meaningful insights.

### 3.5 Concordance across protein expression and morphological features

**Figure 6.**
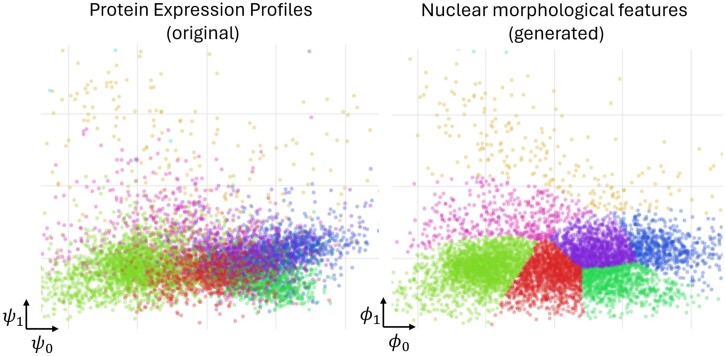
Results of feature space alignment between protein expression and morphological features of generated image patches, revealing a clear link between protein expression and nuclear morphology of generated images. Each plot visualizes individual patches as dots. The left plot shows true protein expression profiles (originally 38D) of tissue spots in a 2D space (*ψ*), while the right plot similarly shows 63 morphological features of nuclei from our generated images (ϕ). There is a one-to-one correspondence between dots across both plots. We applied canonical correlation analysis for dimensionality reduction, with eight distinct colors representing clusters identified by a Gaussian mixture model applied to nuclear features of generated image patches. The consistent clustering between both plots underscores a strong correlation between protein expression and nuclear morphology of generated images, validating our generative model’s effectiveness.

We have further validated the proposed approach through subspace alignment analysis. Figure 6 shows the concordance between true protein expression and morphological features of image patches generated on the basis of these protein expression. This was achieved through canonical correlation analysis (CCA) over the protein expression and morphological feature spaces followed by clustering as discussed in the figure caption. These results clearly show the strong association of nuclear morphological features of generated patches with the underlying protein expression. Similarly, the concordance between the nuclear morphological features of original and the corresponding synthetic patches is depicted in Supplementary Fig. S2 clearly demonstrating the preservation of these characteristics despite multiple sources of information loss.

## 4 Limitations, conclusions, and future work

In conclusion, the Ouroboros framework has shown its effectiveness in simultaneously generating tissue images based on protein expressions and predicting protein expressions from tissue images as well as cross-linking protein expression alterations to routine histology imaging. This proof-of-concept approach is an encouraging step forward in this domain with the potential to advance our understanding of the association of histology and underlying protein expression. Nevertheless, to fully realize and expand the impact of such approaches, extensive validation studies and systematic analyses of the causal implication of perturbation experiments are essential in the future as well as the more technical objectives such as generation of larger tissue sections with histological coherence.

## Supplementary Material

btae399_Supplementary_Data
