# Supplementary material for: Ouroboros: cross-linking protein expression perturbations and cancer histology imaging with generative-predictive modeling
Source: Bioinformatics. 2024 Sep 4;40(Suppl 2):ii174–81. doi: 10.1093/bioinformatics/btae399 (PMC11373318; doi:10.1093/bioinformatics/btae399)
Supplement: btae399_Supplementary_Data [file btae399_supplementary_data.docx]

**Ouroboros: Cross-linking Protein Expression Perturbations and Cancer Histology Imaging with Generative-Predictive Modeling**

Srijay Deshpande^1^ Sokratia Georgaka^2^ Michael Haley^2^ Robert Sellers^2^ James Minshull^5^ Jayakrupakar Nallala^3^ Martin Fergie^4^ Nicholas Stone^3^ Nasir Rajpoot^1^ Syed Murtuza Baker^2^ Mudassar Iqbal^2^ Kevin Couper^2^ Federico Roncaroli^5^ and Fayyaz Minhas^1^

^1^ Tissue Image Analytics Centre, Department of Computer Science, University of Warwick, CV4 7AL, United Kingdom, ^2^ Faculty of Biology, Medicine and Health, University of Manchester, M13 9PL, United Kingdom, ^3^ School of Physics and Astronomy, University of Exeter, EX4 4QL, United Kingdom, ^4^ Division of Informatics, Imaging and Data Science, University of Manchester, M13 9PT, United Kingdom and ^5^ Division of Neuroscience and Experimental Psychology, M13 9PL, United Kingdom ^∗^ Corresponding author: [fayyaz.minhas@warwick.ac.uk](mailto:fayyaz.minhas@warwick.ac.uk)

**Supplementary Materials**

**1. Protein Expression Prediction Results**

| Protein Biomarker | ResNet50 | | Ouroboros | | |
| --- | --- | --- | --- | --- | --- |
|  | Pearson | Spearman | Pearson | Spearman |  |
| cMYC | 0.64 ± 0.14 | 0.45 ± 0.22 | **0.79 ± 0.05** | **0.62 ± 0.17** |  |
| S100B | 0.49 ± 0.14 | 0.38 ± 0.24 | **0.75 ± 0.06** | **0.65 ± 0.11** |  |
| PTEN | 0.57 ± 0.1 | 0.33 ± 0.23 | **0.74 ± 0.10** | 0.51 ± 0.21 |  |
| CDK4 | 0.64 ± 0.03 | 0.55 ± 0.17 | **0.74 ± 0.10** | 0.53 ± 0.31 |  |
| TCIRG1 | 0.55 ± 0.07 | 0.37 ± 0.22 | **0.73 ± 0.14** | 0.45 ± 0.39 |  |
| CD11b | 0.37 ± 0.24 | 0.36 ± 0.23 | **0.72 ± 0.04** | **0.72 ± 0.03** |  |
| MET | 0.56 ± 0.09 | 0.46 ± 0.11 | **0.70 ± 0.19** | 0.54 ± 0.27 |  |
| PDGFRa | 0.57 ± 0.06 | 0.5 ± 0.1 | **0.70 ± 0.15** | 0.51 ± 0.25 |  |
| NESTIN | 0.59 ± 0.06 | 0.59 ± 0.15 | **0.68 ± 0.09** | 0.58 ± 0.18 |  |
| YKL40 | 0.4 ± 0.13 | 0.3 ± 0.12 | **0.66 ± 0.12** | 0.50 ± 0.16 |  |
| TMEM119 | 0.53 ± 0.06 | 0.37 ± 0.17 | **0.64 ± 0.16** | 0.50 ± 0.20 |  |
| MCT4 | 0.3 ± 0.12 | 0.24 ± 0.1 | **0.61 ± 0.12** | 0.42 ± 0.26 |  |
| MHCI | 0.43 ± 0.13 | 0.35 ± 0.19 | 0.56 ± 0.19 | 0.52 ± 0.21 |  |
| pERK | 0.42 ± 0.11 | 0.43 ± 0.08 | 0.41 ± 0.42 | 0.39 ± 0.37 |  |
| SOX2 | 0.3 ± 0.17 | 0.32 ± 0.26 | 0.39 ± 0.36 | 0.32 ± 0.44 |  |
| GFAP | 0.06 ± 0.13 | 0.09 ± 0.19 | 0.39 ± 0.28 | 0.35 ± 0.21 |  |
| OLIG2 | -0.01 ± 0.11 | 0.01 ± 0.1 | 0.39 ± 0.11 | 0.36 ± 0.11 |  |
| KI67 | 0.07 ± 0.13 | 0.08 ± 0.12 | 0.39 ± 0.08 | 0.37 ± 0.09 |  |
| SOX10 | 0.09 ± 0.11 | 0.08 ± 0.1 | 0.35 ± 0.31 | 0.33 ± 0.28 |  |
| P2RY12 | 0.15 ± 0.2 | 0.15 ± 0.2 | 0.30 ± 0.35 | 0.27 ± 0.37 |  |
| SMAa | 0.16 ± 0.07 | 0.1 ± 0.06 | 0.30 ± 0.19 | 0.21 ± 0.10 |  |
| IBA1 | 0.14 ± 0.17 | 0.16 ± 0.18 | 0.29 ± 0.29 | 0.28 ± 0.28 |  |
| CD14 | 0.18 ± 0.21 | 0.22 ± 0.22 | 0.28 ± 0.46 | 0.23 ± 0.47 |  |
| EGFR | 0.34 ± 0.1 | 0.37 ± 0.15 | 0.28 ± 0.43 | 0.22 ± 0.40 |  |
| CD68 | 0.11 ± 0.09 | 0.12 ± 0.08 | 0.28 ± 0.18 | 0.25 ± 0.16 |  |
| CD24 | -0.11 ± 0.06 | -0.1 ± 0.06 | 0.27 ± 0.37 | 0.32 ± 0.31 |  |
| CD74 | -0.0 ± 0.05 | -0.01 ± 0.05 | 0.27 ± 0.24 | 0.24 ± 0.24 |  |
| CD16 | 0.05 ± 0.05 | 0.05 ± 0.05 | 0.27 ± 0.17 | 0.25 ± 0.15 |  |
| CD11c | 0.11 ± 0.1 | 0.12 ± 0.11 | 0.26 ± 0.19 | 0.22 ± 0.22 |  |
| HLADR | 0.13 ± 0.07 | 0.12 ± 0.13 | 0.25 ± 0.35 | 0.16 ± 0.38 |  |
| HIF1a | 0.14 ± 0.15 | 0.11 ± 0.11 | 0.24 ± 0.33 | 0.18 ± 0.27 |  |
| VISTA | 0.03 ± 0.02 | 0.03 ± 0.02 | 0.24 ± 0.13 | 0.25 ± 0.12 |  |
| CD44 | 0.25 ± 0.13 | 0.24 ± 0.07 | 0.23 ± 0.44 | 0.19 ± 0.37 |  |
| CD163 | 0.13 ± 0.12 | 0.17 ± 0.12 | 0.23 ± 0.30 | 0.24 ± 0.28 |  |
| CD206 | 0.14 ± 0.1 | 0.17 ± 0.08 | 0.23 ± 0.25 | 0.23 ± 0.27 |  |
| CD31 | 0.04 ± 0.05 | 0.02 ± 0.03 | 0.22 ± 0.19 | 0.10 ± 0.07 |  |
| DNA3 | 0.13 ± 0.08 | 0.12 ± 0.08 | 0.13 ± 0.37 | 0.09 ± 0.36 |  |
| DNA1 | 0.13 ± 0.09 | 0.12 ± 0.09 | 0.12 ± 0.38 | 0.07 ± 0.38 |  |

Supplementary Table 1: Performance of the predictive model in terms of mean ± standard deviation of Pearson and Spearman correlations between predicted and actual expression of individual proteins across test image patches. The correlations above 0.6 are in bold.

**2. Residual Blocks used in Ouroboros Framework**

**
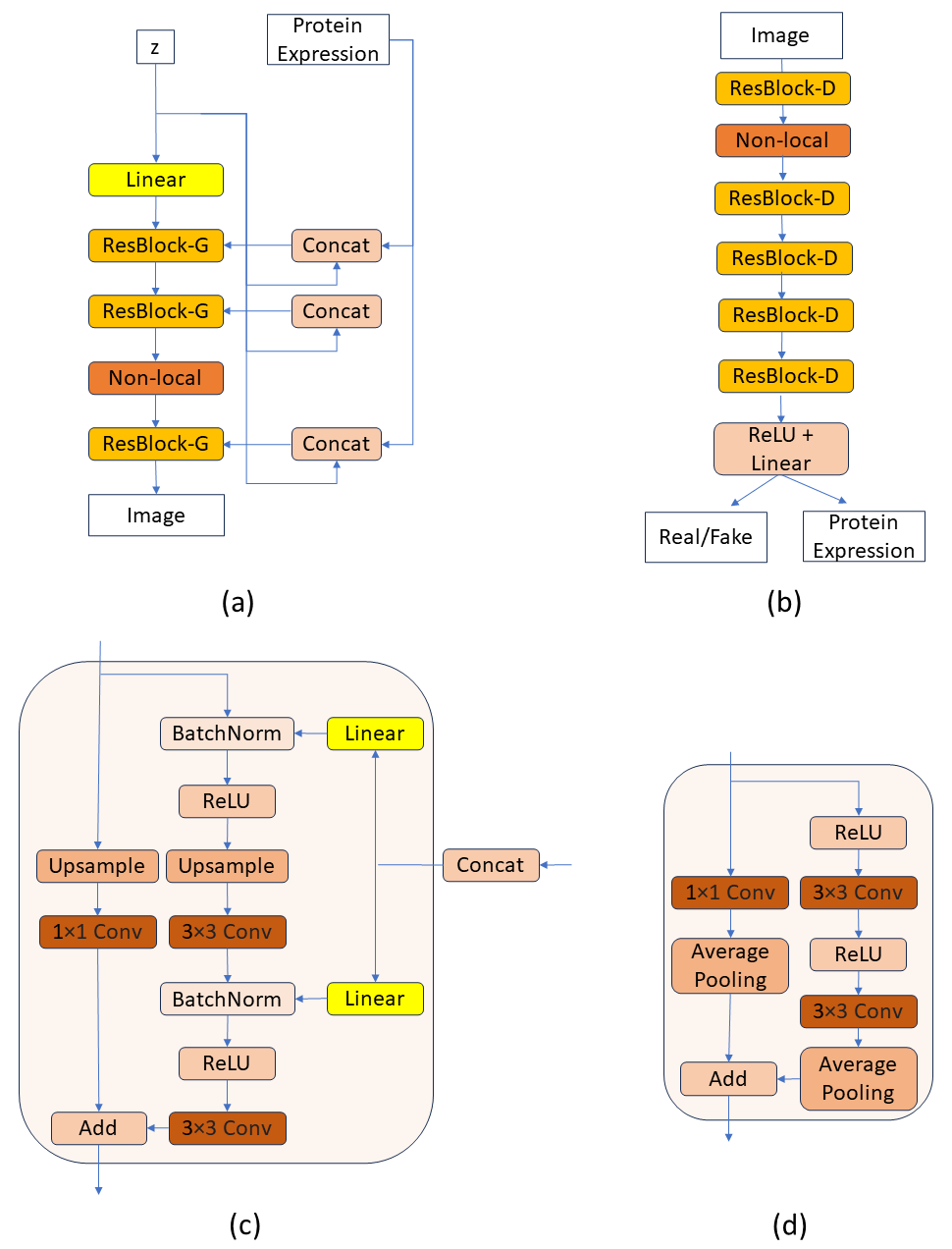
**

Supplementary Figure 1: Residual blocks used in Ouroboros. Blocks (a) and (c) are used in the generator neural network, whereas, blocks (b) and (d) used in the discriminator.

**3. Concordance between the nuclear morphological features of original and the corresponding synthetic patches**


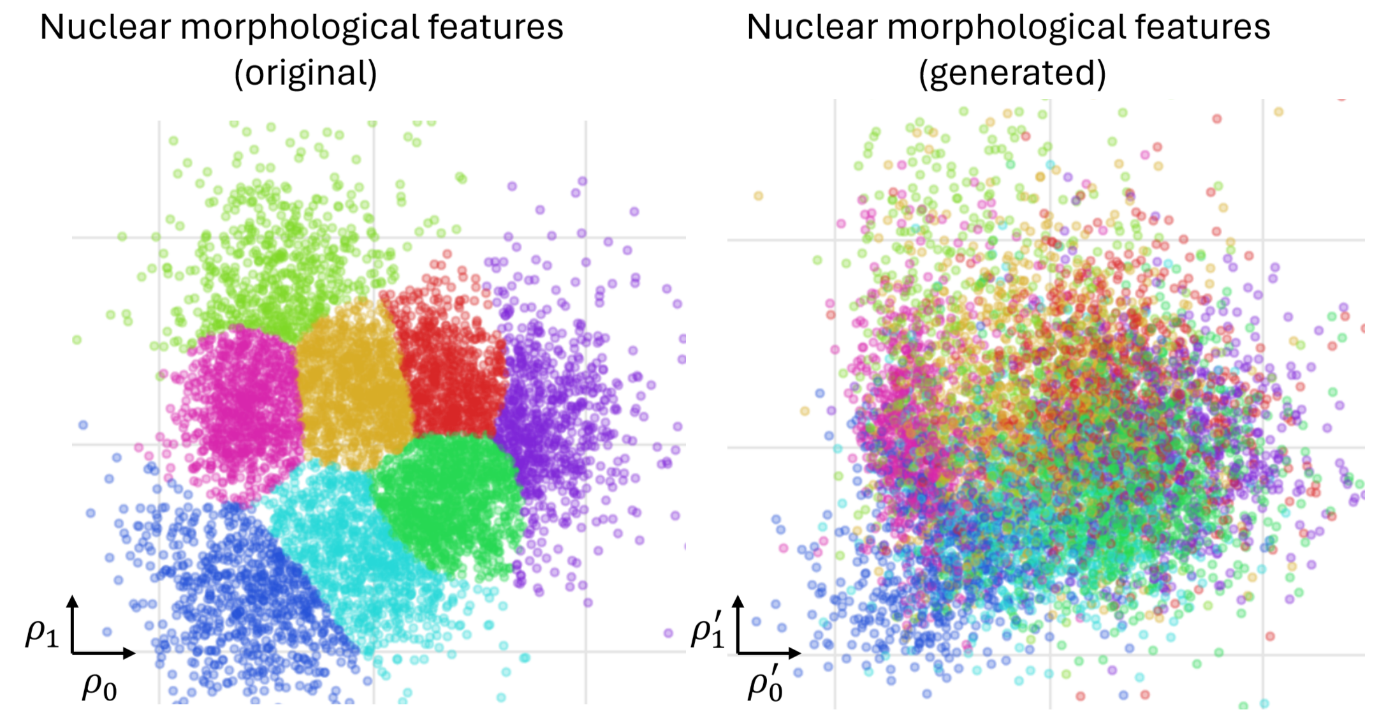


Supplementary Figure 2: Results of feature space alignment between morphological features of true image patches (left) and patches generated from protein expression of corresponding spots during testing, revealing large-scale preservation of underlying nuclear morphology in generative modeling. Each plot visualizes individual patches as dots. The left plot shows nuclear morphological features (originally 63-dimensional) of original tissue patches in a 2D space, while the right plot similarly shows the 63 morphological features of nuclei from our generated images. There is a one-to-one correspondence between dots across both plots. We applied canonical correlation analysis for dimensionality reduction, with eight distinct colors representing clusters identified by a gaussian mixture model applied to nuclear features of original image patches. The consistent clustering between both plots underscores a strong correlation between true and generated nuclear morphologies validating our generative model's effectiveness. Discordances can be expected due to significant averaging and dimensionality reduction in plotting as well as potential errors.

**4. Contributions and Acknowledgements:**

Concept & supervision: FM; Experiments and execution: SD, FM; WSI acquisition and pathology review: FR, JM; Hyperion data acquisition and alignment: KC, MH, RS; Sequencing data analysis support: SG, SMB, MI; Spectroscopic data acquisition: JN, NS; Funding acquisition: SG, MF, NS, NR, SMB, MI, KC, FR, FM (PI). Writeup: SD and FM with review by all authors. We would like to acknowledge the funding support under CLIRPath EPSRC grant EP/W00058X/1, and the University of Manchester Wellcome Institutional Strategic Support Fund grant (204796/Z/16/Z).
